# Tuberculosis during pregnancy in the United States: Racial/ethnic disparities in pregnancy complications and in-hospital death

**DOI:** 10.1371/journal.pone.0194836

**Published:** 2018-03-26

**Authors:** Erika M. Dennis, Yun Hao, Mabella Tamambang, Tasha N. Roshan, Knubian J. Gatlin, Hanane Bghigh, Oladimeji T. Ogunyemi, Fatoumata Diallo, Kiara K. Spooner, Jason L. Salemi, Omonike A. Olaleye, Kashif Z. Khan, Muktar H. Aliyu, Hamisu M. Salihu

**Affiliations:** 1 Texas Southern University, Houston, Texas, United States of America; 2 Department of Family and Community Medicine, Baylor College of Medicine, Houston, Texas, United States of America; 3 Department of Health Policy, Vanderbilt University Medical Center, Nashville, Tennessee, United States of America; University of North Carolina at Chapel Hill School of Medicine, UNITED STATES

## Abstract

**Background:**

Despite decades of efforts to eliminate tuberculosis (TB) in the United States (US), TB still contributes to adverse ill health, especially among racial/ethnic minorities. According to the Centers for Disease Control and Prevention, in 2016, about 87% of the TB cases reported in the US were among racial and ethnic minorities. The objective of this study is to explore the risks for pregnancy complications and in-hospital death among mothers diagnosed with TB across racial/ethnic groups in the US.

**Methods:**

This retrospective cohort study utilized National Inpatient Sample data for all inpatient hospital discharges in the US. We analyzed pregnancy-related hospitalizations and births in the US from January 1, 2002 through December 31, 2014 (n = 57,393,459). Multivariable logistic regression was applied to generate odds ratios for the association between TB status and the primary study outcomes (i.e., pregnancy complications and in-hospital death) across racial/ethnic categories.

**Results:**

The prevalence of TB was 7.1 per 100,000 pregnancy-related hospitalizations. The overall prevalence of pregnancy complications was 80% greater among TB-infected mothers than their uninfected counterparts. Severe pre-eclampsia, eclampsia, placenta previa, post-partum hemorrhage, sepsis and anemia occurred with greater frequency among mothers with a TB diagnosis than those without TB, irrespective of race/ethnicity. The rate of in-hospital death among TB patients was 37 times greater among TB-infected than in non-TB infected mothers (468.8 per 100,000 versus 12.6 per 100,000). A 3-fold increased risk of in-hospital death was observed among black TB-negative mothers compared to their white counterparts. No racial/ethnic disparities in maternal morbidity or in-hospital death were found among mothers with TB disease.

**Conclusion:**

TB continues to be an important cause of morbidity and mortality among pregnant women in the US. Resources to address TB disease should also target pregnant women, especially racial/ethnic minorities who bear the greatest burden of the disease.

## Introduction

Tuberculosis (TB) remains a major public health threat, claiming millions of lives annually throughout the world.[1] “In 2015, there were an estimated 10.4 million new (incident) TB cases worldwide, of which 5.9 million (56%) were among men, 3.5 million (34%) among women and 1.0 million (10%) among children.”[1] According to the Centers for Disease Control and Prevention (CDC), TB adversely affects minority populations, with approximately 87% of the reported TB cases in the United States in 2016 comprised of racial and ethnic minorities.[2] Worldwide, TB is the leading cause of death from a single infectious agent.[3] Moreover, about half a million women die yearly from TB with a disproportionate number dying during their reproductive years.[4–7] A recent systematic review and meta-analysis revealed that active TB in pregnancy is associated with adverse maternal and fetal outcomes.[8] According to the 2017 TB report of the World Health Organization, “of the 10.4 million incident cases of TB globally in 2016, about 1.9 million were attributable to undernourishment, 1.0 million to HIV infection, 0.8 million to smoking and 0.8 million to diabetes”[3]—all of which could influence the association of TB and pregnancy complications and/or maternal mortality in TB-infected pregnant women.

As a result of the sustained public health efforts toward elimination over the previous two decades, the incidence of TB has decreased progressively, and currently stands at about 3.0 cases per 100,000 in the general US population.[9] However, a recent study of TB among pregnant mothers in the US suggests an increase in TB rates associated with elevated levels of pregnancy complications.[10] These findings raise concerns and warrant further investigation. Given the historically cumulative evidence of racial/ethnic disparity in the incidence of TB within the country, we conducted this study with the following objectives: (1) determine disparity in the prevalence of TB among hospitalized pregnant women of different racial/ethnic populations; (2) determine the risk of selected pregnancy complications and in-hospital death in pregnant women infected with tuberculosis across racial/ethnic sub-groups.

## Materials and methods

Our analysis covered the period from January 1, 2002 through December 31, 2014 using cross-sectional data from the Nationwide Inpatient Sample (NIS). The NIS, made available by the Healthcare Cost and Utilization Project (HCUP), currently constitutes the largest all-payer, publicly available inpatient database in the US. Each year, to create the sample of inpatient hospitalizations, HCUP employs a two-stage cluster sampling design that first stratifies all nonfederal community hospitals from participating states by five major hospital characteristics: rural/urban location, number of beds, geographic region, teaching status, and ownership. Then, from each unique stratum, 20% of hospitals are selected using a systematic random sampling technique.[11] In the second stage, all inpatient hospitalizations from designated hospitals in stage 1 are selected for inclusion in the final NIS database. Beginning in 2012, the NIS sampling strategy was changed from keeping all discharges in a sample of hospitals to drawing a sample of discharges from all hospitals. The NIS approximates a 20% systematic sample that is representative of the population of all discharges on critical hospital and patient characteristics.[12] In 2014, 45 states participated in the NIS—up from 35 states in 2002—and each year of the NIS contains approximately 7–8 million hospitalizations (over 35 million when weighted).

Hospitalizations for pregnant women ages 15–49 were identified using the NEOMAT variable, which was created by HCUP to identify maternal discharges on the basis of a wide range of diagnosis and procedure codes documented in the medical record.[11] To assess the study’s primary exposures, we scanned International Classification of Diseases, Ninth Edition, Clinical Modification (ICD-9-CM) codes (the principal diagnosis and up to 24 secondary diagnoses) in each woman’s hospitalization record for an indication of TB status. TB-affected pregnancies are those with one or more of the following codes: 010-018.x (TB); 137.x (sequelae of TB), 647.3 (TB complicating pregnancy/childbirth). We examined the following pregnancy complications, also identified using ICD-9-CM codes, as a composite variable: placenta previa (641.0x, 641.1x), placental abruption (641.2x), placenta accreta (667.0x), other antepartum hemorrhage (641.8x, 641.9x), postpartum hemorrhage (666.0x, 666.1x, 666.2x), sepsis (038.x, 670.2x, 785.52, 995.91, 995.92), and anemia (280–285, 648.2x). In-hospital death was defined as a documented disposition of ‘expired’, indicating death prior to hospital discharge.

Individual-level sociodemographic and behavioral characteristics were also extracted from the NIS databases. Maternal age in years was classified into three categories: 15–24, 25–34, and 35–49. Self-reported maternal race/ethnicity was first based on ethnicity (Hispanic or non-Hispanic [NH]), and the NH group further subdivided by race (white, black, or other). Median household income, which served as a proxy for socioeconomic status, was estimated using the patient’s zip code and subsequently grouped into quartiles. We classified the primary payer for hospital admission into three categories: government (Medicare/Medicaid), private (commercial carriers, private health maintenance organization [HMOs], and preferred provider organization [PPOs]), and other sources (e.g., self-pay and charity). We also considered several hospital characteristics including teaching status (teaching vs. non-teaching), location (urban vs. rural), and US region (Northeast, Midwest, South, or West).

Joinpoint regression was used to estimate and describe temporal changes in the rates of TB and HIV during the 13-year study period. Joinpoint regression is valuable in identifying key periods in time marking changes in the rate of events over time.[13, 14] The iterative model-building process began by fitting the annual rate data to a straight line with no joinpoints, which assumed a single trend best described the data. Then a joinpoint—reflecting a change in the trend—was added to the model and a Monte Carlo permutation test assessed the improvement in model fit. The process continued until a final model with an optimal (best-fitting) number of joinpoints was selected, with each joinpoint indicating a change in the trend, and an annual percent change (APC) estimated to characterize how the rate was changing within each distinct trend segment.

Descriptive statistics including frequencies and percentages were used to describe the distribution of pregnancy-related hospitalizations across patient- and hospital-level characteristics, and stratified by exposure groups (TB disease or no TB). Since national estimates were desired, all statistical analyses were weighted using an HCUP-provided discharge-level weight that accounts for the NRD’s sampling design and appropriately generates variance estimates. Multivariable logistic regression was then used to generate adjusted odds ratios (OR) that quantified the magnitude of the association between infection status and the main outcomes of the study (pregnancy complications and in-hospital death). Statistical analyses were performed with SAS, version 9.4 (SAS Institute, Inc., Cary, NC); we assumed a 5% type I error rate for all hypothesis tests (two-sided). Due to the de-identified, publicly-available nature of NIS data, the analyses performed for this study were considered exempt by the Baylor College of Medicine Institutional Review Board. It is pertinent to mention that some of the results of our analyses contained small numbers which must be suppressed in accordance with guidelines set forth by HCUP. The reason is to prevent possible identification of these individuals, and in such cases, we described the findings in text without displaying the actual values.

## Results

Out of a total of 57,393,459 admissions associated with pregnancy and child birth, 4,053 cases of TB were observed (rate = 7.1 per 100,000 pregnancy-related hospitalizations). In Table 1, we present socio-demographic characteristics of mothers comparing those in the entire population of pregnant women versus those with TB. There was excess representation of women of Hispanic origin, who made up 34.3% of the mothers with TB, although they accounted for only 18.9% of the study population. Black women were second with a relative excess representation in TB-positive women of about 40% (16.9% TB positive vs. 11.9% TB negative). Women of advanced maternal age and in the lowest income category were over-represented among mothers with TB disease. Mothers with public or no health insurance coverage were disproportionately affected with TB disease whereas private health insurance coverage was under-represented among cases of TB. Across the US, excess cases of TB were most pronounced in the Northeast and in urban teaching health facilities.

**Table 1:** Distribution of socio-demographic and hospital characteristics among pregnant women 15–49 years of age, by TB status, United States, 2002–2014.

TB Status			
Characteristic	TB negative (N = 57,389,406) n (%)	TB positive (N = 4,053) n (%)	*P*-value
**Race**			<0.01
NH White	24,134,826 (42.1)	454 (11.2)	
NH Black	6,811,459 (11.9)	686 (16.9)	
Hispanic	10,820,553 (18.9)	1,392 (34.3)	
NH Other	4,912,230 (8.6)	922 (22.7)	
Missing	10,710,338 (18.7)	599 (14.8)	
**Age**			<0.01
15–24	19,438,558 (33.9)	1,245 (30.7)	
25–34	29,523,561 (51.4)	2,081 (51.4)	
35–49	8,427,286 (14.7)	727 (17.9)	
**Income**			<0.01
Lowest	15,583,320 (27.2)	1,480 (36.5)	
2^nd^	14,210,454 (24.8)	970 (23.9)	
3^rd^	13,816,488 (24.1)	851 (21.0)	
Highest	12,702,519 (22.1)	643 (15.9)	
**Insurance**			<0.01
Public	24,690,396 (43.0)	2,541 (62.7)	
Private	28,949,564 (50.4)	956 (23.6)	
Other	3,749,446 (6.5)	556 (13.7)	
**Hospital Region**			<0.01
Northeast	9,465,447 (16.5)	981 (24.2)	
Midwest	12,181,059 (21.2)	729 (18.0)	
South	21,736,246 (37.9)	1,399 (34.5)	
West	14,006,654 (24.4)	943 (23.3)	
**Hospital Type**			<0.01
Rural	6,443,415 (11.2)	194 (4.8)	
Urban, non-teaching	23,148,240 (40.3)	806 (19.9)	
Urban, teaching	27,553,731 (48.0)	3,028 (74.7)	

**Note**: NH = non-Hispanic. Percentages are column percentages used to compare the distribution of that characteristic in hospitalizations for pregnant women who are TB+ and TB-. Percentages may not add to 100% due to missing data. P-value estimated from a Rao-Scott modified chi-square test assessing whether there is statistical association between TB status and each characteristic.

**Fig 1:**
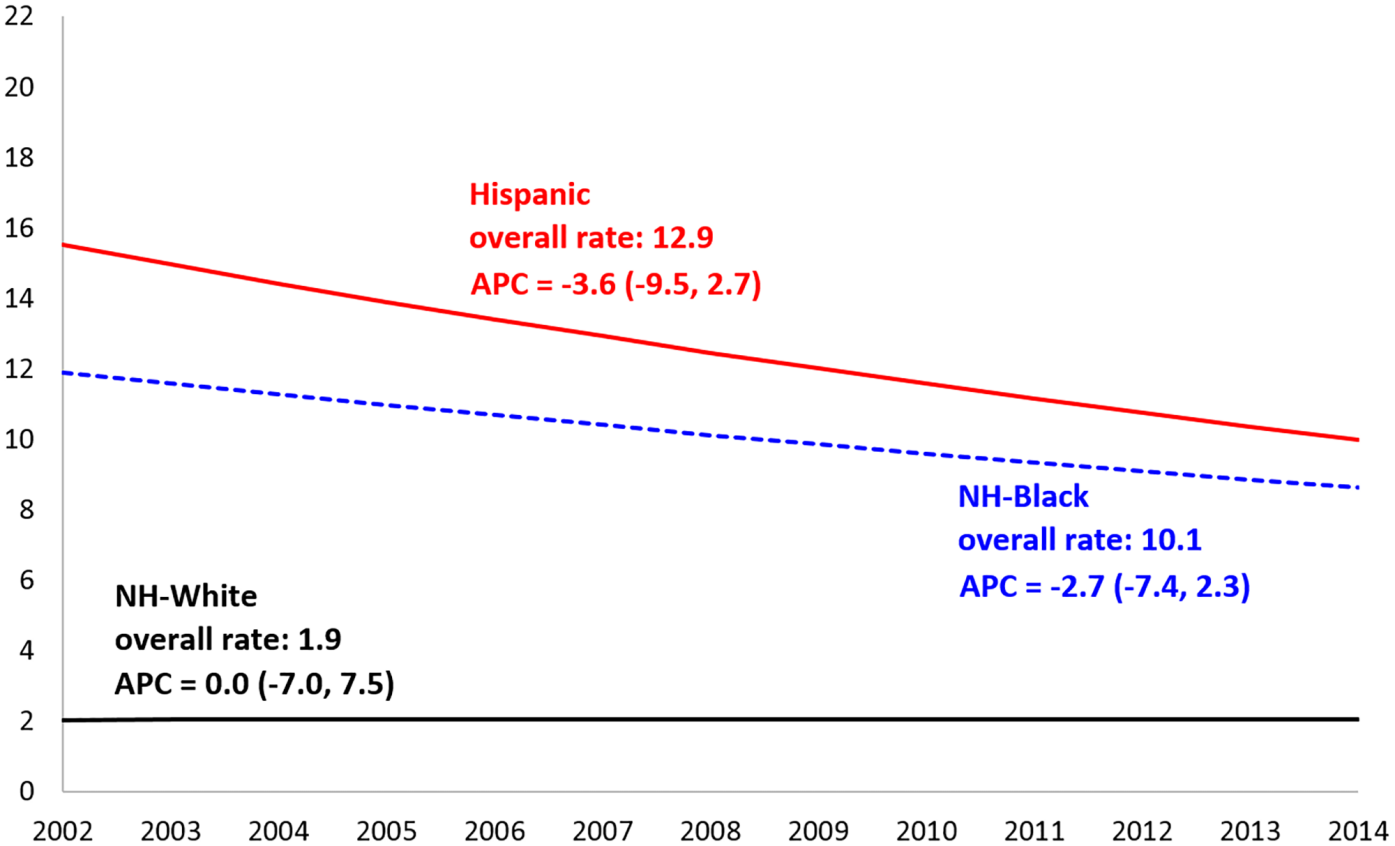
Temporal trends in the prevalence of tuberculosis, by race/ethnicity, in the United States, 2002–2014. The X-axis represents the year of discharge and the Y-axis represents the rate of tuberculosis (per 100,000 pregnancy-related hospitalizations). Lines represent the trend estimated by joinpoint regression. Values represent the annual percent change (APC), point estimate (95% confidence interval). The ‘overall rate’ represents the rate of tuberculosis from January 1, 2002 to December 31, 2014, inclusive.

Fig 1 displays the prevalence of TB among mothers across racial/ethnic groups over the study period. The highest overall rate of TB was among the Hispanic population (12.9 per 100,000) while the lowest rate was among non-Hispanic whites (1.9 per 100,000). Numerically, the prevalence of TB among mothers decreased at an average percent rate of 3.6/100,000 per year and 2.7/100,000 per year among Hispanics and non-Hispanic blacks, respectively. However these rates were not statistically significant. The rates among non-Hispanic whites remained flat throughout the study period.

The rate of selected pregnancy complications among mothers, by TB status, is presented in Table 2. The overall rate of pregnancy complications was 80% greater among TB-infected mothers than their TB-uninfected counterparts. The rate of severe pre-eclampsia, eclampsia, placenta previa, post-partum hemorrhage, sepsis, and anemia was higher among hospitalized mothers with a diagnosis of active TB than in those without such a diagnosis. By contrast, mild pre-eclampsia, placenta accreta, and placental abruption occurred with higher frequency in TB-free than in TB-affected mothers. The most remarkable findings were related to sepsis and eclampsia—in such cases mothers with TB had six- and four-fold prevalence rates, respectively, as compared to TB-free mothers.

**Table 2:** Comparison of the rate (per 1,000 pregnancy-related hospitalizations) of selected pregnancy complications among hospitalized pregnant women aged 15–49 years of age, by TB status, United States, 2002–2014.

TB Status			
Complications ^a^	TB negative (N = 57,389,406) Rate	TB positive (N = 4,053) Rate	Proportionality Ratio ^b^
Any Pre-eclampsia	35.1	35.0	1.0
Mild Pre-eclampsia	22.7	14.0	0.6
Severe Pre-eclampsia	12.5	21.0	1.7
Eclampsia	1.0	3.9	3.9
Placenta accreta	3.3	2.3	0.7
Placental abruption	10.6	8.7	0.8
Placenta previa	5.2	9.7	1.9
Other antepartum hemorrhage	3.6	3.5	1.0
Postpartum hemorrhage	25.8	45.4	1.8
Sepsis	1.3	8.1	6.2
Anemia	102.8	216.4	2.1
**Composite (all)**	**166.5**	**293.2**	**1.8**

^**a**^ For any individual pregnancy complication, no pregnancy-related hospitalization is counted more than once, although women with multiple complications may appear in more than one group. For the composite indicator, hospitalizations for women with multiple complications making up the composite indicator are still counted only once in this group.

^**b**^ Proportionality ratio was derived by dividing the prevalence of the characteristic among TB-positive mothers by the prevalence of the same characteristic in TB-free mothers. A value that is greater than one indicates preponderance of that characteristic among TB mothers. The ratios are approximated to one decimal point.

Table 3 contains a summary of the analysis for the association between race/ethnicity and maternal pregnancy complications as a composite outcome by TB status. Among TB-negative mothers, non-Hispanic blacks experienced almost a 70% greater likelihood for pregnancy complications when compared to their white counterparts 1.67 (1.62, 1.72) while Hispanic mothers had almost a comparable likelihood of pregnancy complications compared to whites 1.07 (1.01, 1.14). Among mothers with a diagnosis of TB, similar risk levels for pregnancy complications as a composite outcome was observed across the three racial/ethnic categories.

**Table 3:** Racial/Ethnic adjusted odds ratios for pregnancy complications^a^ by TB status among pregnant women in the United States, 2002–2014.

**TB-negative**		
**Race/Ethnicity**	**Crude OR (95% CI)**	**Adjusted OR (95% CI)**
NH Whites	1.00	1.00
NH Blacks	1.84 (1.78, 1.89)	1.67 (1.62, 1.72)
Hispanics	1.17 (1.09, 1.24)	1.07 (1.01, 1.14)
**TB-positive**		
**Race/Ethnicity**	**Crude OR (95% CI)**	**Adjusted OR (95% CI)**
NH Whites	1.00	1.00
NH Blacks	0.94 (0.56, 1.60)	0.86 (0.49, 1.49)
Hispanics	0.77 (0.48, 1.23)	0.72 (0.44, 1.17)

OR = odds ratio; CI = confidence interval. Adjusted estimates were derived by loading the following covariates onto the model: for maternal age group, income level, insurance, and hospital region.

^a^ Includes a composite indicator of any of the following adverse pregnancy outcomes: preeclampsia, eclampsia, placenta accreta, placental abruption, placenta previa, other antepartum hemorrhage, postpartum hemorrhage, sepsis, or anemia).

The total number of in-hospital deaths in the entire population of pregnant women was 7,225, equivalent to a rate of 12.6 per 100,000 pregnancy-related hospitalizations. Of these numbers, 7,206 mothers who died were not infected with TB (rate = 12.6 per 100,000 pregnancy-related hospitalizations), while 19 were cases of TB (rate = 468.8 per 100,000 pregnancy-related hospitalizations).

A summary of the results for in-hospital death among mothers with and without TB disease by racial/ethnic composition are shown in Table 4. Among mothers that were TB-negative, the likelihood of death was about three-fold among black mothers as compared to their white counterparts. By contrast, Hispanics had the same mortality risk as whites among TB-negative mothers. Analysis among mothers with TB revealed similar likelihood of mortality for both blacks and whites. Although the likelihood of mortality appeared reduced among Hispanic mothers, the difference was not statistically significant.

**Table 4:** Racial/Ethnic in-hospital death rates (per 100,000 pregnancy-related hospitalizations) and adjusted odds ratios by TB status among pregnant women in the United States, 2002–2014.

**TB-negative**			
**Race/Ethnicity**	**In-hospital death rate**	**Crude OR (95% CI)**	**Adjusted OR (95% CI)**
NH Whites	9.1	1.00	1.00
NH Blacks	29.2	3.20 (2.77, 3.70)	2.77 (2.35, 3.25)
Hispanics	10.2	1.12 (0.94, 1.33)	1.12 (0.94, 1.33)
**TB-positive**			
**Race/Ethnicity**	**In-hospital death rate**	**Crude OR (95% CI)**	**Adjusted OR (95% CI)**
NH Whites	1101.3	1.00	1.00
NH Blacks	1457.7	1.41 (0.12, 16.0)	0.95 (0.10, 9.23)
Hispanics	287.4	0.31 (0.02, 5.33)	0.16 (0.10, 2.69)

OR = odds ratio; CI = confidence interval. Adjusted estimates were derived by loading the following covariates onto the model: for maternal age group, income level, insurance, and hospital region.

## Discussion

Among the three racial/ethnic groups considered in this study (i.e., Whites, Blacks and Hispanics), we found mothers of Hispanic origin to have the highest prevalence, accounting for more than one-third of all TB diagnoses among pregnant women, even though they comprised less than 20% of the entire population of pregnant mothers. Foreign-born persons in the US account for most cases of TB in the US, and are more than 13 times as likely to be diagnosed with TB as US-born individuals.[15] Thus, our finding of TB preponderance among Hispanic pregnant mothers is not surprising and is consistent with the recent 2016 CDC TB Surveillance report which revealed that the Hispanics/Latinos race/ethnicity have a disproportionately higher TB burden compared to Whites and Blacks/African Americans.[2] Again, this is likely a reflection of a significant number of foreign born persons in this population. However, the database used for this analysis did not contain information about nativity (country of birth), and it was therefore impossible to estimate the contribution of foreign-born individuals to the excess cases of TB accounted for by this population sub-group. The absence of information on country of origin also limited our ability to derive the contribution of foreign-born Hispanic mothers to the preponderance of TB cases in that ethnic group.

The prevalence of TB among pregnant women consistently declined throughout the study period. The annual percent decrease was 3.6%, 2.7% and 1.9% among Hispanic, black and white mothers respectively. This result is consistent with reports from the CDC and other sources of a downward trend in TB incidence in the general US population over the period of the study.[16–19] However, a recent publication using the same dataset as ours suggested a rise in TB prevalence among US pregnant women in the period 2003–2011.[10] The authors reported a 9.8% increase in TB rate each year, a finding that is in stark contrast to consistent and reputable reports of persistent decline in TB rates in the US as a result of enhanced TB control measures nationwide. A letter to the editor reacting to that publication suggested that the major flaw in that article was the way the authors defined tuberculosis, which encompassed both clinically confirmed as well as unconfirmed self-reported patients’ history of TB.[19] Inclusion of the latter (using ICD 9 code V12.01) results in gross overestimation of both the prevalence and temporal trends in TB infection among pregnant women in the US.

Our analysis with respect to pregnancy complications and in-hospital death revealed contrasting findings depending on TB status. Among TB-negative mothers, non-Hispanic blacks experienced almost a 70% greater likelihood for pregnancy complications when compared to their white counterparts, while Hispanic mothers had almost a comparable likelihood of pregnancy complications compared to whites. However, in mothers with a diagnosis of TB, similar risk levels for pregnancy complications as a composite outcome was observed across the three racial/ethnic categories. This discordance in racial/ethnic disparity was similarly observed with respect to in-hospital death. Although TB-free black mothers experienced a likelihood of in-hospital death of about three-fold as compared to their white counterparts, this disparity vanished completely among TB-positive mothers. An explanation for these unexpected observations is difficult to advance, and one can only speculate the reasons for these surprising results. The 3-fold elevated risk of death among black TB-negative mothers in comparison to their white counterparts is consistent with findings from the general population of pregnant women as reported by the US nationwide Pregnancy Mortality Surveillance System.[20] Although race/ethnic-specific causes of death did not seem to have shifted over the previous decades based on surveillance data, it will be informative to conduct future studies that delineate in-hospital causes of death stratified by diagnostic codes for each racial/ethnic group. This could provide a broader perspective of underlying causes of maternal mortality that drive black-white disparity among TB-negative mothers.

The lack of black-white disparity observed among mothers with tuberculosis disease with respect to in-hospital death and mortality outcomes could partially be explained by differences in resilience. Resilience is generally defined as the coping capacity or qualities that enable an individual to thrive in the face of adverse conditions.[21] Previous reports have observed a direct relationship between levels of resilience in a population and positive health conditions and the converse also holds.[22, 23] Black-white disparities have been shown to exist, with blacks showing somewhat greater levels of resilience in the face of adverse health events.[24, 25] Based on that premise, one could speculate that cultural differences in the ability to cope with adverse conditions such as tuberculosis could serve as an important modulating factor in precipitating complications of pregnancy or mortality. Although resilience, as a measure, is typically employed in the context of psychosocial research, it could as well apply to ostensibly biological outcomes, such as pregnancy complications or in-hospital death. A limitation in our study, in this respect, is the absence of a measure of resilience that could capture the construct. Another explanation for the findings among mothers with TB disease could be that black mothers had less advanced or severe form of TB which could have dampened the level of pregnancy complications and in-hospital death among them blunting an underlying black-white disparity. However, this argument does not appear tenable since advanced and severe TB is generally more likely among racial/ethnic minorities who suffer from poor access to quality healthcare.[26, 27]

There are other limitations with this study that are worth discussing. The data analyzed did not include information on quality of care received by mothers, data on drug resistant TB as well as data on management options instituted to treat TB. Consequently, it was not possible to assess the role of quality of care in the results being reported for this study. Furthermore, the association of TB and adverse maternal outcomes could be affected by severity of TB disease, site of TB dissemination, drug-resistant TB, other co-morbidities such as HIV and diabetes, timing of TB diagnosis and treatment.[8] Our analysis could not adequately evaluate the various factors mentioned above and treatment schedules for TB patients and how their impact influenced pregnancy complications or in-hospital death in these patients. Despite these shortcomings, our study is one of the largest in the world that examined the relationships among TB status, pregnancy complications and in-hospital death across racial/ethnic sub-populations. The findings in this study highlight the need to allocate more resources to address TB disease among pregnant women especially, racial/ethnic minorities who bear the greatest burden of the disease.
